# The proteasome activity reporter GFP-Cl1 is degraded by autophagy in the aging model *Podospora anserina*

**DOI:** 10.12688/f1000research.5337.1

**Published:** 2014-09-30

**Authors:** Matthias Wiemer, Heinz D. Osiewacz

**Affiliations:** 1 Institute of Molecular Biosciences and Cluster of Excellence Frankfurt Macromolecular Complexes; Department of Biosciences, J W Goethe University, Frankfurt, 60438, Germany

## Abstract

The degradation of damaged proteins is an important vital function especially during aging and stress. The ubiquitin proteasome system is one of the major cellular machineries for protein degradation. Health and longevity are associated with high proteasome activity. To demonstrate such a role in aging of *Podospora anserina*, we first analyzed the transcript and protein abundance of selected proteasome components in wild-type cultures of different age. No significant differences were observed. Next, in order to increase the overall proteasome abundance we generated strains overexpressing the catalytic proteasome subunits PaPRE2 and PaPRE3. Although transcript levels were strongly increased, no substantial effect on the abundance of the corresponding proteins was observed. Finally, the analysis of the *P. anserina* strains expressing the sequence coding for the CL1 degron fused to the *Gfp* gene revealed no evidence for degradation of the GFP-CL1 fusion protein by the proteasome. Instead, our results demonstrate the degradation of the CL1-degron sequence via autophagy, indicating that basal autophagy appears to be a very effective protein quality control pathway in *P. anserina*.

## Introduction

The degradation of proteins, in particular of those that are damaged or are present in excess, is an important vital function of biological systems and is implicated in several cellular processes such as cell cycle control, proliferation, differentiation, apoptosis and protein quality control ^1^. Impairments in protein degradation lead to the formation of protein aggregates ^2, 3^, promote the aging process ^4, 5^ and convey the development of neurodegenerative diseases like Alzheimer’s or Parkinson’s disease ^6^. There are two major pathways involved in protein degradation: autophagy and degradation by the ubiquitin proteasome system (UPS) ^7^. Autophagy is effective in nutrient recycling and protein degradation. During autophagy proteins or whole organelles are engulfed by a double membrane forming autophagosomes that deliver their cargo to the lysosome in animals and the vacuole in plants and fungi ^8^. The UPS consists of a large number of different ubiquitin ligases that act jointly with the proteasome, a multi-protein complex with proteolytic activities. The ubiquitin ligases identify and mark proteins that need to be removed, by formation of a chain of ubiquitin on the target protein ^9^. A ubiquitin chain linked at K-48 is recognized by the 26S proteasome ^10^. The 26S proteasome consists of two subcomplexes, the catalytic 20S core particle and the 19S regulatory particle. The 19S regulatory particle conveys the identification, deubiquitination, unfolding and transport of the substrate into the proteolytic chamber. The core particle is responsible for the degradation of the target proteins. It is composed of four stacked rings, which enclose the proteolytic chamber. The inner rings consist of 7 β-subunits, including the proteolytic active PRE3 (β1), PUP1 (β2) and PRE2 (β5). The three catalytic subunits are the first substrates of the proteasome. Each contains a prosequence that is removed during assembly of the proteasome by an autocatalytic mechanism ^11, 12^. The assembled β-subunits are framed by rings of seven α-subunits, blocking the entrance to the proteolytic chamber, if no regulatory particle is bound (reviewed in: ^13^).

Previous studies revealed that aging reduces the expression of genes coding for proteasome subunits and the activity of the proteasome in several model systems ^14– 16^. Also, several studies indicate a health and lifespan prolonging effects of high proteasome activity. For example, the proteasome activity is elevated in human fibroblast cell cultures derived from centenarians ^14^ and in the liver of the naked mole rat ^17, 18^, a long-living rodent. Moreover, the overexpression of genes coding for proteasome subunit β1 or β5 in human fibroblasts was reported to lead to an increase in overall proteasome abundance and activity, resulting in an increased capacity to cope with stress ^19^. Another component influencing proteasome activity is the proteasome assembly protein UMP1. *Saccharomyces cerevisiae* overexpressing *ScUmp1* shows increased lifespan and viability in response to oxidative stress ^20^. In *S. cerevisiae*, high levels of proteasome subunit ScRPN4 were reported to increase UPS capacity, enhance resistance to proteotoxic stress and increase replicative lifespan ^21^. Overall, it appears that the proteasome is a relevant target for aging research. The data suggest that keeping protease activity high during aging can lead to an increase in the healthy lifespan of biological systems.

We use the filamentous ascomycete *Podospora anserina* as a model organism to investigate the mechanisms of aging including the role of different quality control pathways (for recent reviews see: ^22– 24^). In this study we investigated the impact of protein degradation by the UPS and autophagy. Although we could not demonstrate a role of the UPS, we established that the degradation of GFP-CL1 protein, that was expected to be a target of the proteasome, occurred via autophagy.

## Materials and methods

### *P. anserina* strains and cultivation

*P. anserina* was grown on plates with M2 medium (0.25 g/l KH _2_PO _4_ (Merck Cat# 5099.1000), 0.3 g/l K _2_HPO _4_ (Roth Cat# P749.1), 0.25 g/l MgSO _4_ × 7 H _2_O (Merck Cat# 1.05886.0500), 0.5 g/l urea (Merck Cat# 1.08487.0500) and 10 g/l yellow dextrin (Roth Cat# 6777.1), supplemented with 2.5 mg/l biotin (Serva Cat# 15060), 50 mg/l thiamine (Serva Cat# 36020), 5 mg/l citric acid × 1 H _2_O (Sigma-Aldrich Cat# C-0759), 5 mg/l ZnSO _4_ × 7 H _2_O (Merck Cat# Z-0625), 1 mg/l Fe(NH _4_) _2_(SO _4_) _2_ × 6 H _2_O (Merck Cat# 1.03861.0250), 2.5 mg/l CuSO _4_ × 5 H _2_O (Merck Cat# 2790.1000), 25 mg/l MnSO _4_ × 1 H _2_O (Serva Cat# 28405), 50 mg/l Na _2_MoO _4_ × 2 H _2_O (Serva Cat# 30207) and 50 mg/l H _3_BO _3_ (Merck Cat# 100165.5000) after sterilization of the basal medium) or in shaking Erlenmeyer flasks with CM-Medium (70 mM NH _4_Cl (Merck Cat# 1.01145.5000), 7.3 mM KH _2_PO _4_, (Merck Cat#1.04873.100), 6.7 mM KCl (Merck Cat# 1.04936.1000), 2 mM MgSO _4_ (Merck Cat# 1.05886.0500), 1% glucose (Sigma Cat# G-5400), 0.2% tryptone (Otto Nordwald Cat# 211701), 0.2% yeast extract (DIFCO Cat# 0127-07), 5 mM FeCl _2_ × 7 H _2_O (Merck Cat# 13478-10-9), 3.5 mM ZnSO _4_, (Merck Cat# 108883), 6.2 mM MnCl _2_, (Merck Cat# 5934.0100), pH 6.5) under constant light at 27°C. For germination, spores were incubated for two days in the dark on standard cornmeal agar supplemented with 60 mM ammonium acetate (Merck Cat# 1116.1000) ^25^. Pieces of the mycelium derived from germinated spores were transferred on M2 medium to obtain cultures of specific age.

### Quantitative Real-time PCR (qRT-PCR)

After germination of spores, pieces of the mycelium were directly used or grown at 27° and constant light on M2 medium for 13 – 16 days (middle-aged) or 21 – 24 days (senescent), depending on the lifespan of the specific individual, to obtain cultures of specific age. A piece of the growth front was subsequently spread on a fresh M2 plate covered with cellophane (BioRad Cat# 1650963) and grown for 3 days. RNA was extracted with RNA-Plant kit (Machery-Nagel Cat# 740.949.250) and cDNA synthesis was performed using iScript kit (BioRad Cat# 170-8891). After dilution of cDNA to a concentration of 10 ng/µl, 20 ng was used per qRT-PCR reaction (IQ SybrGreen SuperMix, BioRad cat# 170-8882). The primers summarized in Table 1 were used to perform the qRT-PCR with three technical replicates per sample. A specific culture was compared to the mean CP of the juvenile cultures. Relative expression was normalized to *PaPorin* with the following formula ^26^.

$$\text{ratio}=\frac{{\left(E_{\text{target}}\right)}^{\Delta {\text{CP}}_{{\text{target}}^{\left(\text{control–sample}\right)}}}}{{\left(E_{\text{Porin}}\right)}^{\Delta {\text{CP}}_{{\text{Porin}}^{\left(\text{control–sample}\right)}}}}$$

E = PCR-Efficiency; CP = crossing point

**Table 1.** Primers used for qRT-PCR.

PaNo.	Gene	Primer	Sequence
Pa_5_4560	*PaPre3*	Pre3 for	ATGGAATTCGGTACATCGGG
		Pre3 rev	GAGGATAACGCCGTCTTTGA
Pa_1_12250	*PaPre2*	rTPre2for	TTGTTCACAGAGCAGGAGCAG
		rTPre2rev	CGATCTTGATGGGGCAGT
Pa_1_24350	*PaUmp1*	ump1for	TCAACCGAACTCCGATACTC
		ump1rev2	TGGCCTCCCACTGTTTTAAG

### Western blot analysis

To obtain total protein extracts, fungi of specific age were spread on a cellophane foil covered M2 surface for 3 days. Proteins were extracted as described in ^24^. Briefly, the mycelia were harvested, ground under liquid nitrogen, mixed with extraction buffer and centrifuged at 14,000 g at 4°C for 10 min. The supernatant was recovered and used for the experiments. The protein extracts were fractionated by 2-phase SDS-PAGE (14% separating gels) according to standard protocol ^27^. Proteins were subsequently transferred to PVDF membranes (Millipore Cat# IPFL00010). Blocking, antibody incubation and washing was performed according to western blot analysis handbook (LIC-OR Bioscience, Bad Homburg, Germany). The following primary antibodies were used: anti-PaPRE2 (rabbit, 1:500 dilution, raised against the specific peptide [H]-WKTKLEKGEFSNVT-[OH]; Sigma), anti-PaPRE3 (1:2500 dilution, raised against the specific synthetic peptide [H]-LYLPDTDYKVRHEN-[OH]; Sigma), anti-PaPUP1 (rabbit, 1:5000 dilution, raised against the specific peptide Ac-CLKRNYIKPNERT-amid, NEP), anti-HSP60 (mouse, 1:4000 dilution, Biomol, Cat# SPA-807), Anti-GFP (mouse, 1:10000 dilution, Sigma-Aldrich Cat# G6795 RRID:AB_563117). Secondary antibodies conjugated with IRDye 680 (1:15000 dilution, goat anti-mouse 680RD: LI-COR Biosciences Cat# 926-68070 RRID:AB_10956588) or IRDye CW 800 (1:15000 dilution, goat anti-rabbit 800: LIC-OR Biosciences, Cat# 926-3221) were used. After western transfer, the polyacrylamid gels were stained 1 h with coomassie blue as additional loading control.

### Cloning procedures and generation of *P. anserina* mutants

The vector pExMthph ^28^ was used as backbone for the generation of *PaPre2*, *PaPre3* and *PaUmp1* overexpression plasmids. For the assembly of pPaPre2Ex1, pExMthph was cut with BamHI and XbaI. The *PaPre2* gene and terminator were amplified with the primers Pre2ExpFor (AA GGATCCATGGACACCCTCGTTGCG; restriction sites are underlined) and Pre2ExpXbaRev (AA AGATCTTGGCCCTCCTTACTAGAC), cut with BamHI and XbaI and ligated with the backbone. For the generation of pPaPre3Ex1, the *PaPre3* gene and terminator were amplified with the primers PaPre3FwdBam (TT GGATCCATGGAATTCGGTACATCGGG) and PaPre3RevPst (TT CTGCAGCCCACAACCAGAACCTTTCAC) cut with BamHI and PstI and ligated with the similarly restricted vector pExMthph. pPaUmp1Ex1 was generated by amplification of *PaUmp1*, including the terminator, with the primers PaUmp1FwdBamHI (TT GGATCCATGGTAAGTTGCAGCCAACC) and PaUmp1RevPst1 (TT CTGCAGGCTCCCGTGAGGGCAGGAC), restriction of the product with BamHI and PstI, and ligation into the similarly cut vector pExMthph. The generation of a *Gfp-Cl1* overexpression plasmid was performed by 3 fragment ligation. The *Gpd* promotor from *Aspergillus nidulans*, the e *Gfp* gene and the first part of the Cl1-sequence were amplified by PCR with the plasmid pSM5 (based on pSM2 ^29^) as template and with primers eGfp-Pgpd-cl1for (CCTCGAGGTCGACGGTATCGAT AAGCTTGATATCGAATT) containing a HindIII restriction site and eGfp-Pgpd-cl1rev (GTGGCT AGC *GCTGCTGAACCAGTTCTTGCAGGC* CTTGTACAGCTCGTCCAT), containing half of the Cl1 sequence (italic) optimized for codon usage of *P. anserina* and a Eco47III restriction site. The second half of the Cl1-sequence was amplified with the *TrpC* terminator in a similar manner with the primers Ttrpc-cl1for (CTTCAGC GAG *CTCAGCCACTTCGTCATCCACCTCTA* ATCCACTTAACGTTACTGA) containing an Eco53kI restriction sites (underlined) and Ttrpc-cl1rev (CCACCGCGGTGGCGGCCGCTCTAGAAAGAAGGATTACCTC) containing a XbaI restriction site. The PCR products were ligated into the purified backbone of pSM5, previously cut with HindIII and XbaI. The plasmids were used to transform *P. anserina* wild-type spheroplasts according to ^24^. pSM5 was used to generate a strain expressing *Gfp* without degron sequence. Briefly, mycelium of wild type “s” was blended and the cell wall digested with an enzyme solution. After filtration and concentration of sphaeroblasts by centrifugation, the sphaeroblasts were mixed with 10 µg plasmid DNA. Subsequently, polyethylene glycole (Serva Cat# 33136) was added to the sphaeroblasts. Transformants were selected for hygromycin B (Calbiochem Cat# 400051) resistance and the number of integrations was verified by Southern blot analysis.

### Fluorescence microscopy

A piece of 2 day old mycelium was grown on a glass slide with a piece of PASM-medium ^27^ covered with a coverslip and incubated at 27°C for 2 days. Heat stressed samples were incubated at 27°C for 24 h followed by an incubation at 37°C for 24 h. The cover slip with hyphae on it was visualized using a fluorescence microscope (DM LB, Leica, Wetzlar, Germany) with the appropriate excitation and emission filter to detect the GFP signal and a digital camera system (DC500, Leica, Wetzlar, Germany).

## Results

### Regulation of proteasome components during aging and oxidative stress

**Figure 1.**
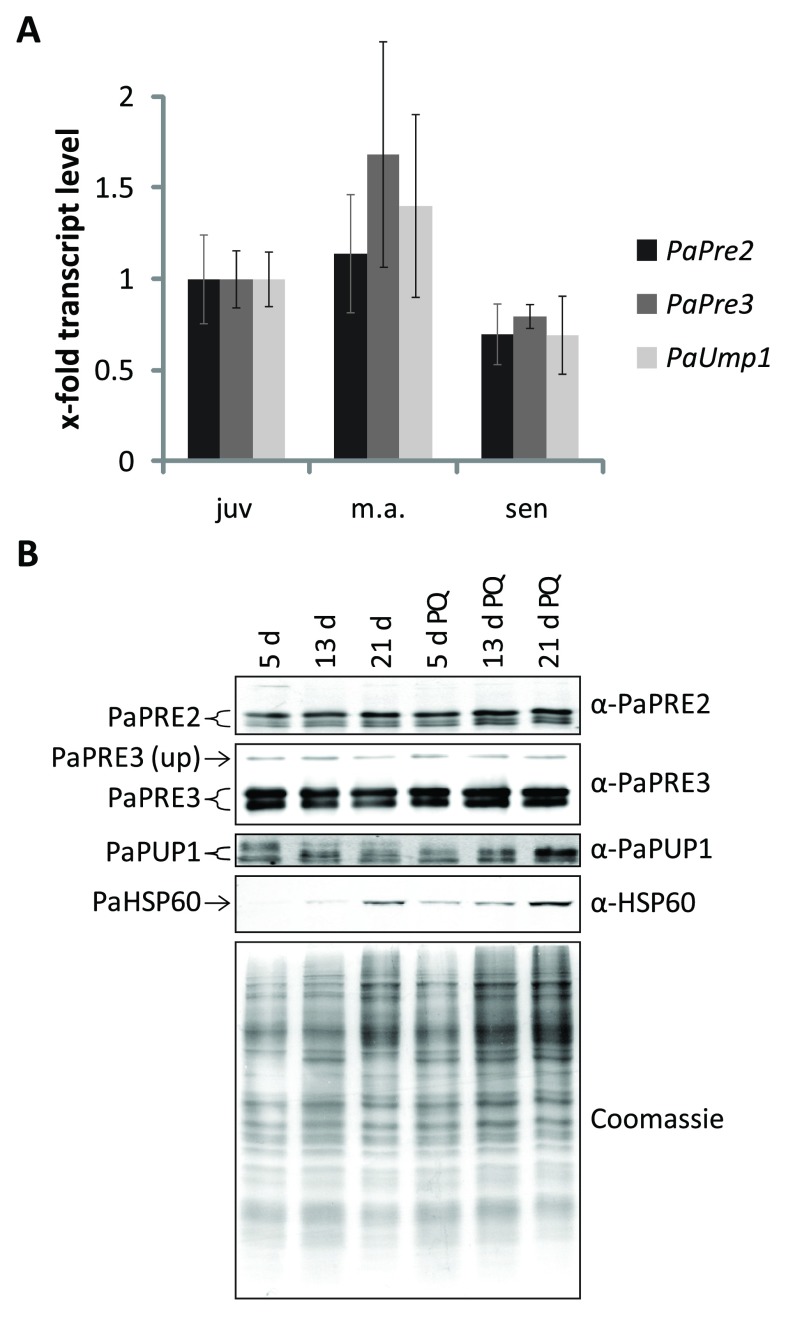
Gene expression and protein abundance of proteasome subunits during aging and PQ-stress. ( **A**) The expression of *PaPre2*, *PaPre3* and *PaUmp1* transcripts in juvenile, middle-aged and senescent samples is depicted relative to the juvenile wild type as mean ± SEM (5 – 7 biological replicates). ( **B**) Western blot analysis of 50 µg total protein extracts of 5 d, 13 d and 21 d old wild type cultures grown on medium with and without the addition of 5 µM paraquat. Used antibodies are indicated on the right. The polyacrylamid gel stained with coomassie after blotting is used as loading control.

In order to address the role of the UPS on aging of *P. anserina*, we investigated the expression of the genes coding for proteolytic subunits *PaPre2* (β5) and *PaPre3* (β1) and of the proteasome assembly factor *PaUmp1.* First, we determined the abundance of transcript by using total RNA of juvenile, middle-aged and senescent cultures ( Figure 1A). No significant changes in mRNA levels were observed in cultures of different age although mRNA abundance of all three genes was slightly reduced in senescent cultures. Next we analyzed protein levels of proteasome subunits in cultures of different age grown in standard growth medium and in medium to which paraquat was added as an inducer of oxidative stress. An increased abundance of mitochondrial HSP60 verified an increase in oxidative stress in senescent and in paraquat treated cultures ( Figure 1B). However, no changes in the abundance of subunits PaPRE2, PaPRE3 and PaPUP1 (β3) of the proteasome were observed in the corresponding *P. anserina* cultures. We thus were unable to demonstrate a role of the ubiquitin proteasome system in counteracting adverse effects on cellular proteins in aged cultures and in cultures challenged with exogenous oxidative stress.

### Overexpression of *PaPre2* or *PaPre3* does not increase the total amount of proteasome

**Figure 2.**
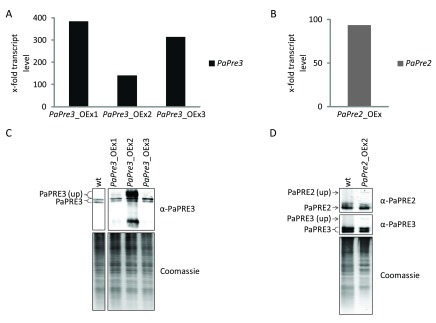
Overexpression of catalytic 20S subunits *PaPre2* or *PaPre3* does not alter the level of processed protein. The expression of *PaPre3* ( **A**) and *PaPre2* ( **B**) in the respective overexpression strain was examined by qRT-PCR. ( **C, D**) Total protein extracts of *PaPre3* (50 µg) and *PaPre2* (60 µg) overexpression strains were probed with α-PaPRE3 and α-PaPRE2 for the amount of processed proteasome subunits. The polyacrylamid gel stained with coomassie after blotting is displayed as loading control.

High activity of the proteasome has been linked to increased health and lifespan ^30– 33^. In human cell cultures, the overexpression of the subunits β5 and β1 was found to increase the overall abundance of the proteasome, as well as its activity and resistance to oxidative stress ^34^. To investigate whether or not such an effect is also observed in *P. anserina*, strains overexpressing the homolog subunits *PaPre2* (β5) and *PaPre3* (β1) were generated. First, plasmids conveying *PaPre2* and *PaPre3* overexpression were constructed and transformed into *P. anserina* spheroplasts ( Table 2). Subsequently, overexpression of the genes was verified by qRT-PCR. *PaPre3* expression was increased by factor 140 to 380 in the respective overexpression strain compared to wild type ( Figure 2A). The *PaPre2* overexpression strain exhibited a 94 times higher *PaPre2* expression than the wild type ( Figure 2B). In the next step, we evaluated protein levels in the overexpression strains by western blot immunodetection. The analysis of three independent *PaPre3* overexpressors revealed two strains with unchanged protein abundance and one strain ( *PaPre3_*OEx2) in which increased PaPRE3 signals occurred ( Figure 2C). However, the detected signals are larger or smaller than expected for processed PaPRE3 and probably represent unprocessed PaPRE3 and a degradation product. A strain overexpressing *PaPre2* showed no increase in PaPRE2 abundance compared to the wild type ( Figure 2D). Thus, despite the strong increase in mRNA abundance, no substantial change in protein levels of the two investigated proteasome subunits was observed in the generated strains.

**Table 2.** Number of ectopic integrations.

Strain	Number of integrations
*Gfp*	2
*Gfp-Cl1*-1	1
*Gfp-Cl1*-2	1
*PaPre2*_OEx	1
*PaPre3*_OEx1	1
*PaPre3*_OEx2	3
*PaPre3*_OEx3	2

### The proteasome activity reporter GFP-CL1 is degraded by autophagy

The activity of the UPS is not exclusively defined by the abundance of proteasome subunits but influenced by various factors like ubiquitin ligases, deubiquitin ligases, ATP-level, the regulatory particle, oxidative stress and post-translational modifications. In order to evaluate the efficiency of the UPS during aging of *P. anserina*, we generated two *Gfp-cl1* strains with similar properties and a *Gfp* strain. Successful transformation of wild type was verified by Southern blot analysis ( Table 2). The introduced genes are under the control of the constitutive *Gpd* promoter of *Aspergillus nidulans. Gfp-cl1* codes for a protein containing the CL1 degron sequence fused to GFP. The CL1 sequence is a part of the *Saccharomyces cerevisiae* genome. It was first described in a ScURA3-CL1 fusion protein, which is unstable in wild type, but stable in strains lacking the ubiquitin ligases *ScUbc6* and *ScUbc7.* Due to these characteristics the Cl1 degron has been used to monitor proteasome activity in various species including fly ^35^, mouse ^36^, rat ^37, 38^ and human cell cultures ^39– 44^.

**Figure 3.**
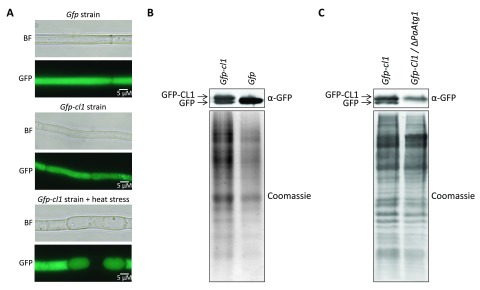
GFP-Cl1 is degraded by autophagy. ( **A**) Fluorescence microscopy analysis of *Gfp* and *Gfp*- *cl1*-1 mutant. Fungi were grown for 2d at 27°. Heat stressed samples were incubated at 37°C for the last 24 h. ( **B**) Western-blot analysis of 60 µg total protein extracts from *Gfp-Cl1*-1 and *Gfp* strains. Proteins were detected by western-blot-analysis with α-GFP antibody. The corresponding polyacrylamid gel was stained with coomassie after blotting as loading control. ( **C**) Western-blot analysis of 45 µg total protein extracts from *Gfp-cl1*-2 strains and from a *Gfp-cl1*-2/Δ *PaAtg1* double mutant. Proteins were detected by western-blot-analysis with α-GFP antibody. The polyacrylamid gel was stained with coomassie after blotting.

In our work, we investigated the degradation of the CL1 degron fused to GFP. Fluorescence microscopy revealed diffuse fluorescence in whole cells of strains expressing *Gfp-cl1* and *Gfp*, respectively, indicating a cytoplasmic localization ( Figure 3A). Significantly, after applying heat stress, the *Gfp-cl1* strain revealed a vacuolar localization of the GFP signal in some parts of the mycelium ( Figure 3A). Western-blot analysis revealed two distinct GFP signals in *Gfp-cl1*-strains ( Figure 3B). One signal corresponds to a protein with a size of 28.8 kDa expected for GFP-CL1 fusion protein while the other has the size of free GFP (26.9 KDa). This result was surprising, because proteasomal degradation should result in total decomposition of GFP-CL1 and provided a first clue for the degradation of the CL1 degron sequence by autophagy since the GFP part remains and is not, or only slowly, degraded by vacuolar proteases ^17, 18^. To verify the degradation of the CL1 degron by autophagy, we generated a *P. anserina* strain lacking *PaAtg1* ^45^ and expressing *Gfp-cl1* by crossing of single mutants and selection of the double mutant. PaATG1 is necessary for autophagy and Δ *PaAtg1*-strains are not able to transport proteins to the vacuole for degradation ^45^. Western blot analysis revealed that the double mutant contains only GFP-CL1 and no free GFP ( Figure 3C), demonstrating that GFP-CL1 is at least partially degraded via autophagy in the wild type of *P. anserina*. This conclusion is supported by the accumulation of green fluorescence in the vacuoles of *Gfp-cl1* overexpressing strains after the induction of autophagy by heat stress ( Figure 3A).

Raw data of qRT-PCR and western blot analyses of proteasome subunits and GFP-CL1 degradation in Podospora anserina Dataset 1 : Raw data of qRT-PCR analysis of the PaPre3 gene used in Figure 1A CP values of the reference gene PaPorin and of the target gene PaPre3 are displayed for juvenile middle-aged and senescent samples
Dataset 2 : Raw data of qRT-PCR analysis of the PaPre2 gene used in Figure 1A CP values of the reference gene PaPorin and of the target gene PaPre2 are displayed for juvenile middle-aged and senescent samples
Dataset 3 : Raw data of qRT-PCR analysis of the PaUmp1 gene used in Figure 1A CP values of the reference gene PaPorin and of the target gene PaUmp1 are displayed for juvenile, middle-aged and senescent samples
Dataset 4 : Raw data of qRT-PCR analysis of the PaPre3 gene used in Figure 2A CP values of the reference gene PaPorin and of the target gene PaPre3 are displayed. The wild type CP is the mean CP value of juvenile samples displayed in Dataset 1
Dataset 5 : Raw data of qRT-PCR analysis of the PaPre2 gene used in Figure 2B CP values of the reference gene PaPorin and of the target gene PaPre2 are displayed. The wild type CP is the mean CP value of the juvenile samples displayed in Dataset 2
Dataset 6: Raw data of western blot displayed in Figure 1B probed with α-PaPRE2. Fluorescence was detected at 700 nm and 800 nm. Both signals are merged in the displayed image. Green signal represents fluorescence at 800 nm generated by anti-rabbit 800 antibody bound to α-PaPRE2. Red signal represents fluorescence at 700 nm. Lane 1 (from left to right): Thermo Fischer PageRulerTM (Cat# 26616) Prestained protein ladder. Lanes 2 – 7: Samples described in Figure 1B.
Dataset 7: Raw data of western blot displayed in Figure 1B probed with α-PaPRE3. Fluorescence was detected at 700 nm and 800 nm. Both signals are merged in the displayed image. Green signal represents fluorescence at 800 nm generated by anti-rabbit 800 antibody bound to α-PaPRE3. Red signal represents fluorescence at 700 nm. Lane 7 (from left to right): Thermo Fischer PageRulerTM Prestained protein ladder. Lanes 1 – 6: Samples described in Figure 1B.
Dataset 8: Raw data of western blot displayed in Figure 1B probed with α-PaPUP1. Fluorescence was detected at 700 nm and 800 nm. Both signals are merged in the displayed image. Green signal represents fluorescence at 800 nm generated by anti-rabbit 800 antibody bound to α-PaPUP1. Red signal represents fluorescence at 700 nm. Lane 10 (from left to right): Thermo Fischer PageRulerTM Prestained protein ladder. Lanes 1 – 6: Samples described in Figure 1B. Lanes 7 - 9 are not relevant to this study.
Dataset 9: Raw data of western blot displayed in Figure 1B probed with α-PaHSP60. Fluorescence was detected at 700 nm. Red signal represents fluorescence at 700 nm generated by anti-mouse 700 antibody bound to α-PaHSP60. Lane 1 (from left to right): Thermo Fischer PageRulerTM Prestained protein ladder. Lanes 2 - 7: Samples described in Figure 1B.
Dataset 10: Raw data of western blot displayed in Figure 2C probed with α-PaPRE3. Fluorescence was detected at 700 nm and 800 nm. Both signals are merged in the displayed image. Green signal represents fluorescence at 800 nm generated by anti-rabbit 800 antibody bound to α-PaPRE3. Red signal represents fluorescence at 700 nm. Lane 1 (from left to right): Thermo Fischer PageRulerTM Prestained protein ladder. Lanes 3 – 5 and lane 9: Samples described in Figure 1C. The other lanes are not relevant to this study.
Dataset 11: Raw data of western blot displayed in Figure 2D probed with α-PaPRE2. Fluorescence was detected at 700 nm and 800 nm. Both signals are merged in the displayed image. Green signal represents fluorescence at 800 nm generated by anti-rabbit 800 antibody bound to α-PaPRE2. Red signal represents fluorescence at 700 nm. Lane 1 (from left to right): Thermo Fischer PageRulerTM Prestained protein ladder. Lanes 3 and 4: Samples described in Figure 1D. Lane 2 is not relevant to this study.
Dataset 12: Raw data of western blot displayed in Figure 2D probed with α-PaPRE3. Fluorescence was detected at 700 nm and 800 nm. Both signals are merged in the displayed image. Green signal represents fluorescence at 800 nm generated by anti-rabbit 800 antibody bound to α-PaPRE3. Red signal represents fluorescence at 700 nm. Lane 1 (from left to right): Thermo Fischer PageRulerTM Prestained protein ladder. Lanes 3 and 4: Samples described in Figure 1D. Lane 2 is not relevant to this study.
Dataset 13: Raw data of western blot displayed in Figure 3B probed with α-GFP. Fluorescence was detected at 700 nm. Red signal represents fluorescence at 700 nm generated by anti-mouse 700 antibody bound to α-GFP. Lane 1 (from left to right): Thermo Fischer PageRulerTM Prestained protein ladder. Lanes 9 and 10: Samples described in Figure 3B.
Dataset 14: Raw data of western blot displayed in Figure 3C probed with α-GFP. Fluorescence was detected at 700 nm. Red signal represents fluorescence at 700 nm generated by anti-mouse 700 antibody bound to α-GFP. Lane 1 (from left to right): Thermo Fischer PageRulerTM Prestained protein ladder. Lanes 5 and 6: Samples described in Figure 3C.

## Discussion

In the current study, we investigated the role of the proteasome in aging of *P. anserina*. Contrary to the mammalian model systems, we did not detect significant reduction of transcript or protein levels of proteasome subunits during aging ^46^. Moreover, attempts to modulate the abundance of selected proteasomal subunits failed, although transcript abundance was strongly increased in the generated overexpression strains. It appears that in *P. anserina* the biosynthesis of the investigated proteasome subunits is under a strong post-transcriptional control.

One aim of our study was the development of an assay to study proteasomal activity. In other systems such assays are based on the microscopic monitoring of fluorescence changes resulting from the degradation of a reporter protein, termed degron, which is fused to GFP. The degron becomes rapidly ubiquitinated and subsequently the whole fusion protein is delivered to the proteasome were it is degraded. A widely used degron is CL1 derived from *S. cerevisiae* and consisting of 15 hydrophobic amino acids. Although this sequence was successfully used to detect proteasome activity in a wide range of organisms including yeast ^47^, fly ^35^, mouse ^36^, rat ^37, 38^ and human cell cultures ^39– 44^, our experiments did not reveal a clear degradation of the whole GFP-CL1 fusion protein as it would be expected for degradation by the proteasome. Beside other reasons, it may be that CL1 is not recognized by the *P. anserina* ubiquitination system and thus does not constitute a functional degron. On the other hand, the sequence may be recognized by both the UPS and the autophagy machinery. Under the investigated conditions autophagy may be by far more efficient than ubiquitination and proteasomal degradation. An overlap of UPS and autophagy substrates has been shown previously ^48– 51^. The degradation of this reporter by autophagy may indeed be a severe problem for the establishment of a reporter gene based proteasome activity assay in filamentous fungi because they seem to be characterized by high level of basal autophagy. In *Aspergillus oryzea*, mitochondria, peroxisomes and nuclei of basal hyphae are degraded during normal growth in an autophagy dependent manner to use the nutrients to support growth ^52^. Previous work in *P. anserina* also detected a high basal autophagy level under non-starved standard growth conditions ^45^. On the other hand, basal autophagy in yeast and mammalian cell cultures appears to be low ^53, 54^. Another complication of the system may be that, as previously shown in *Caenorhabditis elegans* and neuronal rat cells, CL1 fused to GFP can form toxic aggregates if the expression level exceeds the capacity of the degradation system ^55^. In our experiments this latter problem appears not to be valid since the fluorescence signal is distributed throughout the cell although we detected small condensed signals in some cells, which could indicate the formation of protein aggregates. Since such aggregates were only very small spots compared to those demonstrated in the mentioned studies with *C. elegans* and rat cells, the formation of toxic GFP-CL1 aggregates appears to be negligible under the chosen expression conditions.

## Data availability

***figshare:*** Raw data of qRT-PCR and western blot analyses of proteasome subunits and GFP-CL1 degradation in *Podospora anserine*. DOI: 10.6084/m9.figshare.1177910 ^56^
