# Supplementary figures and images for: The proteasome activity reporter GFP-Cl1 is degraded by autophagy in the aging model Podospora anserina
Source: F1000Res. 2014 Sep 30;3:230. [Version 1] doi: 10.12688/f1000research.5337.1 (PMC4264638; doi:10.12688/f1000research.5337.1)

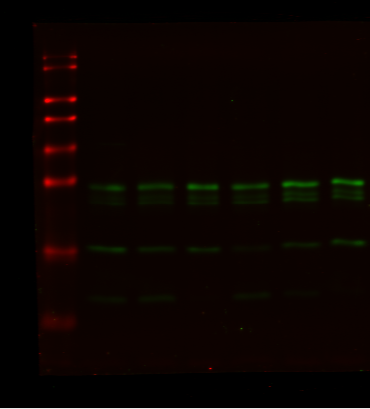

Supplement: Raw data of qRT-PCR and western blot analyses of proteasome subunits and GFP-CL1 degradation in Podospora anserina — Dataset 1 : Raw data of qRT-PCR analysis of the PaPre3 gene used in Figure 1A CP values of the reference gene PaPorin and of the target gene PaPre3 are displayed for juvenile middle-aged and senescent samples Dataset 2 : Raw data of qRT-PCR analysis of the PaPre2 gene used in Figure 1A CP values of the reference gene PaPorin and of the target gene PaPre2 are displayed for juvenile middle-aged and senescent samples Dataset 3 : Raw data of qRT-PCR analysis of the PaUmp1 gene used in Figure 1A CP values of the reference gene PaPorin and of the target gene PaUmp1 are displayed for juvenile, middle-aged and senescent samples Dataset 4 : Raw data of qRT-PCR analysis of the PaPre3 gene used in Figure 2A CP values of the reference gene PaPorin and of the target gene PaPre3 are displayed. The wild type CP is the mean CP value of juvenile samples displayed in Dataset 1 Dataset 5 : Raw data of qRT-PCR analysis of the PaPre2 gene used in Figure 2B CP values of the reference gene PaPorin and of the target gene PaPre2 are displayed. The wild type CP is the mean CP value of the juvenile samples displayed in Dataset 2 Dataset 6: Raw data of western blot displayed in Figure 1B probed with α-PaPRE2. Fluorescence was detected at 700 nm and 800 nm. Both signals are merged in the displayed image. Green signal represents fluorescence at 800 nm generated by anti-rabbit 800 antibody bound to α-PaPRE2. Red signal represents fluorescence at 700 nm. Lane 1 (from left to right): Thermo Fischer PageRulerTM (Cat# 26616) Prestained protein ladder. Lanes 2 – 7: Samples described in Figure 1B. Dataset 7: Raw data of western blot displayed in Figure 1B probed with α-PaPRE3. Fluorescence was detected at 700 nm and 800 nm. Both signals are merged in the displayed image. Green signal represents fluorescence at 800 nm generated by anti-rabbit 800 antibody bound to α-PaPRE3. Red signal represents fluorescence at 700 nm. Lane 7 (from left to right): Thermo Fischer PageRulerTM Prestained protein ladder. La [file f1000research-3-5697-s0000.tgz › Dataset6.tif]

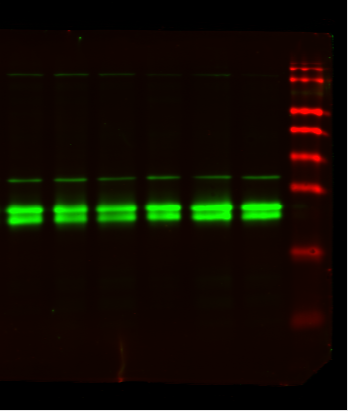

Supplement: Raw data of qRT-PCR and western blot analyses of proteasome subunits and GFP-CL1 degradation in Podospora anserina — Dataset 1 : Raw data of qRT-PCR analysis of the PaPre3 gene used in Figure 1A CP values of the reference gene PaPorin and of the target gene PaPre3 are displayed for juvenile middle-aged and senescent samples Dataset 2 : Raw data of qRT-PCR analysis of the PaPre2 gene used in Figure 1A CP values of the reference gene PaPorin and of the target gene PaPre2 are displayed for juvenile middle-aged and senescent samples Dataset 3 : Raw data of qRT-PCR analysis of the PaUmp1 gene used in Figure 1A CP values of the reference gene PaPorin and of the target gene PaUmp1 are displayed for juvenile, middle-aged and senescent samples Dataset 4 : Raw data of qRT-PCR analysis of the PaPre3 gene used in Figure 2A CP values of the reference gene PaPorin and of the target gene PaPre3 are displayed. The wild type CP is the mean CP value of juvenile samples displayed in Dataset 1 Dataset 5 : Raw data of qRT-PCR analysis of the PaPre2 gene used in Figure 2B CP values of the reference gene PaPorin and of the target gene PaPre2 are displayed. The wild type CP is the mean CP value of the juvenile samples displayed in Dataset 2 Dataset 6: Raw data of western blot displayed in Figure 1B probed with α-PaPRE2. Fluorescence was detected at 700 nm and 800 nm. Both signals are merged in the displayed image. Green signal represents fluorescence at 800 nm generated by anti-rabbit 800 antibody bound to α-PaPRE2. Red signal represents fluorescence at 700 nm. Lane 1 (from left to right): Thermo Fischer PageRulerTM (Cat# 26616) Prestained protein ladder. Lanes 2 – 7: Samples described in Figure 1B. Dataset 7: Raw data of western blot displayed in Figure 1B probed with α-PaPRE3. Fluorescence was detected at 700 nm and 800 nm. Both signals are merged in the displayed image. Green signal represents fluorescence at 800 nm generated by anti-rabbit 800 antibody bound to α-PaPRE3. Red signal represents fluorescence at 700 nm. Lane 7 (from left to right): Thermo Fischer PageRulerTM Prestained protein ladder. La [file f1000research-3-5697-s0000.tgz › Dataset7.tif]

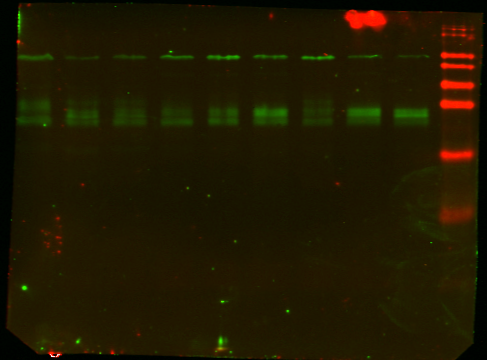

Supplement: Raw data of qRT-PCR and western blot analyses of proteasome subunits and GFP-CL1 degradation in Podospora anserina — Dataset 1 : Raw data of qRT-PCR analysis of the PaPre3 gene used in Figure 1A CP values of the reference gene PaPorin and of the target gene PaPre3 are displayed for juvenile middle-aged and senescent samples Dataset 2 : Raw data of qRT-PCR analysis of the PaPre2 gene used in Figure 1A CP values of the reference gene PaPorin and of the target gene PaPre2 are displayed for juvenile middle-aged and senescent samples Dataset 3 : Raw data of qRT-PCR analysis of the PaUmp1 gene used in Figure 1A CP values of the reference gene PaPorin and of the target gene PaUmp1 are displayed for juvenile, middle-aged and senescent samples Dataset 4 : Raw data of qRT-PCR analysis of the PaPre3 gene used in Figure 2A CP values of the reference gene PaPorin and of the target gene PaPre3 are displayed. The wild type CP is the mean CP value of juvenile samples displayed in Dataset 1 Dataset 5 : Raw data of qRT-PCR analysis of the PaPre2 gene used in Figure 2B CP values of the reference gene PaPorin and of the target gene PaPre2 are displayed. The wild type CP is the mean CP value of the juvenile samples displayed in Dataset 2 Dataset 6: Raw data of western blot displayed in Figure 1B probed with α-PaPRE2. Fluorescence was detected at 700 nm and 800 nm. Both signals are merged in the displayed image. Green signal represents fluorescence at 800 nm generated by anti-rabbit 800 antibody bound to α-PaPRE2. Red signal represents fluorescence at 700 nm. Lane 1 (from left to right): Thermo Fischer PageRulerTM (Cat# 26616) Prestained protein ladder. Lanes 2 – 7: Samples described in Figure 1B. Dataset 7: Raw data of western blot displayed in Figure 1B probed with α-PaPRE3. Fluorescence was detected at 700 nm and 800 nm. Both signals are merged in the displayed image. Green signal represents fluorescence at 800 nm generated by anti-rabbit 800 antibody bound to α-PaPRE3. Red signal represents fluorescence at 700 nm. Lane 7 (from left to right): Thermo Fischer PageRulerTM Prestained protein ladder. La [file f1000research-3-5697-s0000.tgz › Dataset8.tif]

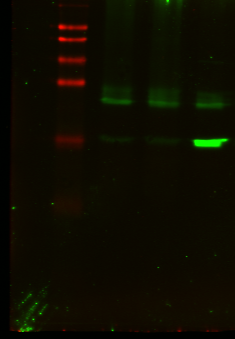

Supplement: Raw data of qRT-PCR and western blot analyses of proteasome subunits and GFP-CL1 degradation in Podospora anserina — Dataset 1 : Raw data of qRT-PCR analysis of the PaPre3 gene used in Figure 1A CP values of the reference gene PaPorin and of the target gene PaPre3 are displayed for juvenile middle-aged and senescent samples Dataset 2 : Raw data of qRT-PCR analysis of the PaPre2 gene used in Figure 1A CP values of the reference gene PaPorin and of the target gene PaPre2 are displayed for juvenile middle-aged and senescent samples Dataset 3 : Raw data of qRT-PCR analysis of the PaUmp1 gene used in Figure 1A CP values of the reference gene PaPorin and of the target gene PaUmp1 are displayed for juvenile, middle-aged and senescent samples Dataset 4 : Raw data of qRT-PCR analysis of the PaPre3 gene used in Figure 2A CP values of the reference gene PaPorin and of the target gene PaPre3 are displayed. The wild type CP is the mean CP value of juvenile samples displayed in Dataset 1 Dataset 5 : Raw data of qRT-PCR analysis of the PaPre2 gene used in Figure 2B CP values of the reference gene PaPorin and of the target gene PaPre2 are displayed. The wild type CP is the mean CP value of the juvenile samples displayed in Dataset 2 Dataset 6: Raw data of western blot displayed in Figure 1B probed with α-PaPRE2. Fluorescence was detected at 700 nm and 800 nm. Both signals are merged in the displayed image. Green signal represents fluorescence at 800 nm generated by anti-rabbit 800 antibody bound to α-PaPRE2. Red signal represents fluorescence at 700 nm. Lane 1 (from left to right): Thermo Fischer PageRulerTM (Cat# 26616) Prestained protein ladder. Lanes 2 – 7: Samples described in Figure 1B. Dataset 7: Raw data of western blot displayed in Figure 1B probed with α-PaPRE3. Fluorescence was detected at 700 nm and 800 nm. Both signals are merged in the displayed image. Green signal represents fluorescence at 800 nm generated by anti-rabbit 800 antibody bound to α-PaPRE3. Red signal represents fluorescence at 700 nm. Lane 7 (from left to right): Thermo Fischer PageRulerTM Prestained protein ladder. La [file f1000research-3-5697-s0000.tgz › Dataset11.tif]

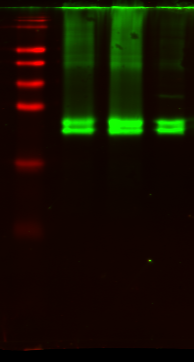

Supplement: Raw data of qRT-PCR and western blot analyses of proteasome subunits and GFP-CL1 degradation in Podospora anserina — Dataset 1 : Raw data of qRT-PCR analysis of the PaPre3 gene used in Figure 1A CP values of the reference gene PaPorin and of the target gene PaPre3 are displayed for juvenile middle-aged and senescent samples Dataset 2 : Raw data of qRT-PCR analysis of the PaPre2 gene used in Figure 1A CP values of the reference gene PaPorin and of the target gene PaPre2 are displayed for juvenile middle-aged and senescent samples Dataset 3 : Raw data of qRT-PCR analysis of the PaUmp1 gene used in Figure 1A CP values of the reference gene PaPorin and of the target gene PaUmp1 are displayed for juvenile, middle-aged and senescent samples Dataset 4 : Raw data of qRT-PCR analysis of the PaPre3 gene used in Figure 2A CP values of the reference gene PaPorin and of the target gene PaPre3 are displayed. The wild type CP is the mean CP value of juvenile samples displayed in Dataset 1 Dataset 5 : Raw data of qRT-PCR analysis of the PaPre2 gene used in Figure 2B CP values of the reference gene PaPorin and of the target gene PaPre2 are displayed. The wild type CP is the mean CP value of the juvenile samples displayed in Dataset 2 Dataset 6: Raw data of western blot displayed in Figure 1B probed with α-PaPRE2. Fluorescence was detected at 700 nm and 800 nm. Both signals are merged in the displayed image. Green signal represents fluorescence at 800 nm generated by anti-rabbit 800 antibody bound to α-PaPRE2. Red signal represents fluorescence at 700 nm. Lane 1 (from left to right): Thermo Fischer PageRulerTM (Cat# 26616) Prestained protein ladder. Lanes 2 – 7: Samples described in Figure 1B. Dataset 7: Raw data of western blot displayed in Figure 1B probed with α-PaPRE3. Fluorescence was detected at 700 nm and 800 nm. Both signals are merged in the displayed image. Green signal represents fluorescence at 800 nm generated by anti-rabbit 800 antibody bound to α-PaPRE3. Red signal represents fluorescence at 700 nm. Lane 7 (from left to right): Thermo Fischer PageRulerTM Prestained protein ladder. La [file f1000research-3-5697-s0000.tgz › Dataset12.tif]

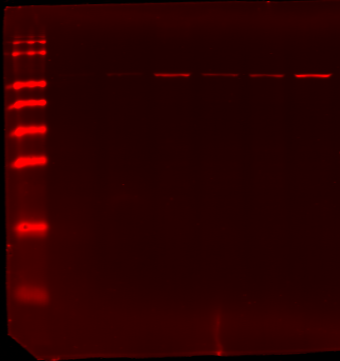

Supplement: Raw data of qRT-PCR and western blot analyses of proteasome subunits and GFP-CL1 degradation in Podospora anserina — Dataset 1 : Raw data of qRT-PCR analysis of the PaPre3 gene used in Figure 1A CP values of the reference gene PaPorin and of the target gene PaPre3 are displayed for juvenile middle-aged and senescent samples Dataset 2 : Raw data of qRT-PCR analysis of the PaPre2 gene used in Figure 1A CP values of the reference gene PaPorin and of the target gene PaPre2 are displayed for juvenile middle-aged and senescent samples Dataset 3 : Raw data of qRT-PCR analysis of the PaUmp1 gene used in Figure 1A CP values of the reference gene PaPorin and of the target gene PaUmp1 are displayed for juvenile, middle-aged and senescent samples Dataset 4 : Raw data of qRT-PCR analysis of the PaPre3 gene used in Figure 2A CP values of the reference gene PaPorin and of the target gene PaPre3 are displayed. The wild type CP is the mean CP value of juvenile samples displayed in Dataset 1 Dataset 5 : Raw data of qRT-PCR analysis of the PaPre2 gene used in Figure 2B CP values of the reference gene PaPorin and of the target gene PaPre2 are displayed. The wild type CP is the mean CP value of the juvenile samples displayed in Dataset 2 Dataset 6: Raw data of western blot displayed in Figure 1B probed with α-PaPRE2. Fluorescence was detected at 700 nm and 800 nm. Both signals are merged in the displayed image. Green signal represents fluorescence at 800 nm generated by anti-rabbit 800 antibody bound to α-PaPRE2. Red signal represents fluorescence at 700 nm. Lane 1 (from left to right): Thermo Fischer PageRulerTM (Cat# 26616) Prestained protein ladder. Lanes 2 – 7: Samples described in Figure 1B. Dataset 7: Raw data of western blot displayed in Figure 1B probed with α-PaPRE3. Fluorescence was detected at 700 nm and 800 nm. Both signals are merged in the displayed image. Green signal represents fluorescence at 800 nm generated by anti-rabbit 800 antibody bound to α-PaPRE3. Red signal represents fluorescence at 700 nm. Lane 7 (from left to right): Thermo Fischer PageRulerTM Prestained protein ladder. La [file f1000research-3-5697-s0000.tgz › Dataset9.tif]

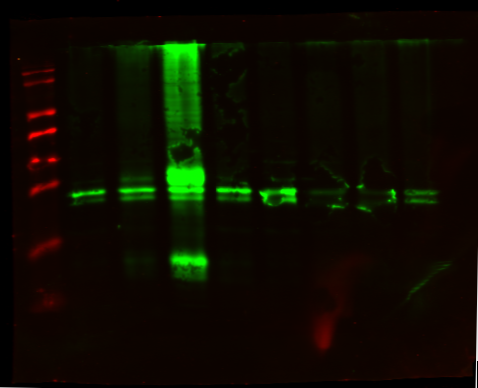

Supplement: Raw data of qRT-PCR and western blot analyses of proteasome subunits and GFP-CL1 degradation in Podospora anserina — Dataset 1 : Raw data of qRT-PCR analysis of the PaPre3 gene used in Figure 1A CP values of the reference gene PaPorin and of the target gene PaPre3 are displayed for juvenile middle-aged and senescent samples Dataset 2 : Raw data of qRT-PCR analysis of the PaPre2 gene used in Figure 1A CP values of the reference gene PaPorin and of the target gene PaPre2 are displayed for juvenile middle-aged and senescent samples Dataset 3 : Raw data of qRT-PCR analysis of the PaUmp1 gene used in Figure 1A CP values of the reference gene PaPorin and of the target gene PaUmp1 are displayed for juvenile, middle-aged and senescent samples Dataset 4 : Raw data of qRT-PCR analysis of the PaPre3 gene used in Figure 2A CP values of the reference gene PaPorin and of the target gene PaPre3 are displayed. The wild type CP is the mean CP value of juvenile samples displayed in Dataset 1 Dataset 5 : Raw data of qRT-PCR analysis of the PaPre2 gene used in Figure 2B CP values of the reference gene PaPorin and of the target gene PaPre2 are displayed. The wild type CP is the mean CP value of the juvenile samples displayed in Dataset 2 Dataset 6: Raw data of western blot displayed in Figure 1B probed with α-PaPRE2. Fluorescence was detected at 700 nm and 800 nm. Both signals are merged in the displayed image. Green signal represents fluorescence at 800 nm generated by anti-rabbit 800 antibody bound to α-PaPRE2. Red signal represents fluorescence at 700 nm. Lane 1 (from left to right): Thermo Fischer PageRulerTM (Cat# 26616) Prestained protein ladder. Lanes 2 – 7: Samples described in Figure 1B. Dataset 7: Raw data of western blot displayed in Figure 1B probed with α-PaPRE3. Fluorescence was detected at 700 nm and 800 nm. Both signals are merged in the displayed image. Green signal represents fluorescence at 800 nm generated by anti-rabbit 800 antibody bound to α-PaPRE3. Red signal represents fluorescence at 700 nm. Lane 7 (from left to right): Thermo Fischer PageRulerTM Prestained protein ladder. La [file f1000research-3-5697-s0000.tgz › Dataset10.tif]

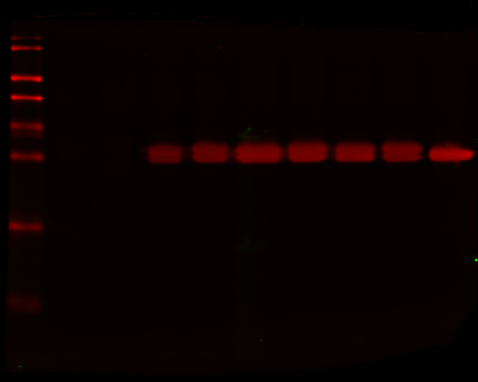

Supplement: Raw data of qRT-PCR and western blot analyses of proteasome subunits and GFP-CL1 degradation in Podospora anserina — Dataset 1 : Raw data of qRT-PCR analysis of the PaPre3 gene used in Figure 1A CP values of the reference gene PaPorin and of the target gene PaPre3 are displayed for juvenile middle-aged and senescent samples Dataset 2 : Raw data of qRT-PCR analysis of the PaPre2 gene used in Figure 1A CP values of the reference gene PaPorin and of the target gene PaPre2 are displayed for juvenile middle-aged and senescent samples Dataset 3 : Raw data of qRT-PCR analysis of the PaUmp1 gene used in Figure 1A CP values of the reference gene PaPorin and of the target gene PaUmp1 are displayed for juvenile, middle-aged and senescent samples Dataset 4 : Raw data of qRT-PCR analysis of the PaPre3 gene used in Figure 2A CP values of the reference gene PaPorin and of the target gene PaPre3 are displayed. The wild type CP is the mean CP value of juvenile samples displayed in Dataset 1 Dataset 5 : Raw data of qRT-PCR analysis of the PaPre2 gene used in Figure 2B CP values of the reference gene PaPorin and of the target gene PaPre2 are displayed. The wild type CP is the mean CP value of the juvenile samples displayed in Dataset 2 Dataset 6: Raw data of western blot displayed in Figure 1B probed with α-PaPRE2. Fluorescence was detected at 700 nm and 800 nm. Both signals are merged in the displayed image. Green signal represents fluorescence at 800 nm generated by anti-rabbit 800 antibody bound to α-PaPRE2. Red signal represents fluorescence at 700 nm. Lane 1 (from left to right): Thermo Fischer PageRulerTM (Cat# 26616) Prestained protein ladder. Lanes 2 – 7: Samples described in Figure 1B. Dataset 7: Raw data of western blot displayed in Figure 1B probed with α-PaPRE3. Fluorescence was detected at 700 nm and 800 nm. Both signals are merged in the displayed image. Green signal represents fluorescence at 800 nm generated by anti-rabbit 800 antibody bound to α-PaPRE3. Red signal represents fluorescence at 700 nm. Lane 7 (from left to right): Thermo Fischer PageRulerTM Prestained protein ladder. La [file f1000research-3-5697-s0000.tgz › Dataset13.tif]

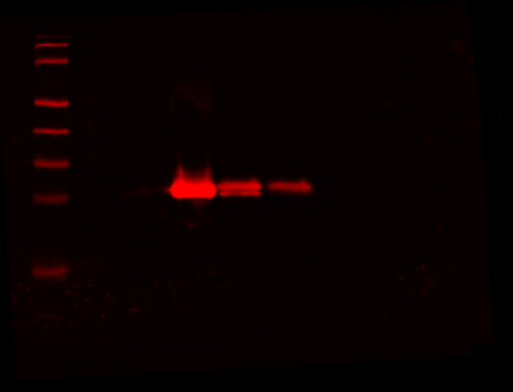

Supplement: Raw data of qRT-PCR and western blot analyses of proteasome subunits and GFP-CL1 degradation in Podospora anserina — Dataset 1 : Raw data of qRT-PCR analysis of the PaPre3 gene used in Figure 1A CP values of the reference gene PaPorin and of the target gene PaPre3 are displayed for juvenile middle-aged and senescent samples Dataset 2 : Raw data of qRT-PCR analysis of the PaPre2 gene used in Figure 1A CP values of the reference gene PaPorin and of the target gene PaPre2 are displayed for juvenile middle-aged and senescent samples Dataset 3 : Raw data of qRT-PCR analysis of the PaUmp1 gene used in Figure 1A CP values of the reference gene PaPorin and of the target gene PaUmp1 are displayed for juvenile, middle-aged and senescent samples Dataset 4 : Raw data of qRT-PCR analysis of the PaPre3 gene used in Figure 2A CP values of the reference gene PaPorin and of the target gene PaPre3 are displayed. The wild type CP is the mean CP value of juvenile samples displayed in Dataset 1 Dataset 5 : Raw data of qRT-PCR analysis of the PaPre2 gene used in Figure 2B CP values of the reference gene PaPorin and of the target gene PaPre2 are displayed. The wild type CP is the mean CP value of the juvenile samples displayed in Dataset 2 Dataset 6: Raw data of western blot displayed in Figure 1B probed with α-PaPRE2. Fluorescence was detected at 700 nm and 800 nm. Both signals are merged in the displayed image. Green signal represents fluorescence at 800 nm generated by anti-rabbit 800 antibody bound to α-PaPRE2. Red signal represents fluorescence at 700 nm. Lane 1 (from left to right): Thermo Fischer PageRulerTM (Cat# 26616) Prestained protein ladder. Lanes 2 – 7: Samples described in Figure 1B. Dataset 7: Raw data of western blot displayed in Figure 1B probed with α-PaPRE3. Fluorescence was detected at 700 nm and 800 nm. Both signals are merged in the displayed image. Green signal represents fluorescence at 800 nm generated by anti-rabbit 800 antibody bound to α-PaPRE3. Red signal represents fluorescence at 700 nm. Lane 7 (from left to right): Thermo Fischer PageRulerTM Prestained protein ladder. La [file f1000research-3-5697-s0000.tgz › Dataset14.tif]
