# Supplementary material for: The proteasome activity reporter GFP-Cl1 is degraded by autophagy in the aging model Podospora anserina
Source: F1000Res. 2014 Sep 30;3:230. [Version 1] doi: 10.12688/f1000research.5337.1 (PMC4264638; doi:10.12688/f1000research.5337.1)
Supplement: Peer review history

# Peer review: Referee response for version 1

**DOI:** 10.5256/f1000research.5697.r6891

**Published:** 2014-12-09

**Authors:** Thorsten Hoppe, Martina Liebl

**Affiliations:** 1 Department of C elegans Genetics and Development, University of Cologne, Cologne, Germany

It is well established that proteasomal degradation declines during aging in different cell culture systems and model organisms. Therefore, it is of general interest whether such activity changes are conserved throughout evolution. Here, the authors analyzed mRNA levels of the 20S proteasomal core subunits PaPre2, PaPre3, and the proteasomal assembly factor PaUmp1 as well as protein levels of PaPRE2, PaPRE3, and PaPUP1 in the fungus *P. anserina*. The experiments are convincing and well described. Surprisingly, the authors did not find any age-dependent differences with respect to the investigated proteasomal factors. However, as measuring proteasomal activity directly did not work, it remained unclear whether a decline in protein turnover is directly linked to aging in this model organism. As highlighted by the authors, other factors including ubiquitin ligases, deubiquitylation enzymes, the 19S regulatory particle, or post-translational modifications of these factors might be even more limiting for ubiquitin-dependent degradation and regulated with age in the fungus. In this context the authors could have discussed the finding that in *C. elegan*s and human stem cells overexpression of the regulatory subunit RPN-6 is sufficient to reduce the amount of toxic protein aggregates and increase life span (Vilchez *et al*. 2012).

The authors state that protein levels of PaPUP1 did not change during aging and paraquat treatment. However, western blot analysis of figure 1B shows increased PaPUB1 levels in 21 day old cultures that were treated with paraquat compared to either paraquat treated young cultures or untreated old cultures. This does not necessarily reflect an age- or stress-dependent increase in PaPUB1 levels in *P. anserina*, but could be explained by a selective survival of fungi with higher PaPUB1 levels under toxic stress conditions.

This study nicely confirms the impression that *P. anserine* exhibits high basal rates of autophagy in contrast to other organisms and thus might serve as an excellent model organism for studying the role of autophagy under different physiological conditions. Given the elevated level of autophagy, this organism however might not be optimal for the investigation of age-dependent changes in the ubiquitin/proteasome system that is relevant for higher organisms.

We have read this submission. We believe that we have an appropriate level of expertise to confirm that it is of an acceptable scientific standard.

# Peer review: Referee response for version 1

**DOI:** 10.5256/f1000research.5697.r6542

**Published:** 2014-12-03

**Authors:** Suresh Rattan

**Affiliations:** 1 Department of Molecular Biology and Genetics, Aarhus University, Aarhus, Denmark

This is a very well written paper dealing with the determination of the proteasome activity in the ageing model Podospora. The experimental work is technically sound and is performed diligently. Although the results obtained, especially with respect to the lack of change in the protesomal activity during ageing of Podospora, appear to be contrary to the ones reported for mammalian systems and for Drosophila, it does not decrease the significance of this study which once again underlies the fact that age-related changes can be both universal (public) or specific (private). Actually, this is the important point that needs to be discussed in this paper as to why such differences are observed (see some papers by George Martin on public and private mechanisms of ageing). Authors should therefore comment on the usefulness and limitations of using model systems such as Podosopora in the context of modulating human ageing and longevity, and for testing/screening potential ageing-modulatory strategies. Furthermore, it will be useful if the implications of enhanced autophagy-related pathways in the context of ageing are discussed a bit more.

On a minor note, just to be up-to-date with the literature on proteasome activity during ageing of various systems (refs 14-16), authors may include and cite a paper on Drosophila ( Hansen *et al., *2012) which deals with the sex-specific differences in proteasome activity changes during ageing etc.

I have read this submission. I believe that I have an appropriate level of expertise to confirm that it is of an acceptable scientific standard, however I have significant reservations, as outlined above.

# Peer review: Referee response for version 1

**DOI:** 10.5256/f1000research.5697.r6268

**Published:** 2014-10-14

**Authors:** Nektarios Tavernarakis

**Affiliations:** 1 Institute of Molecular Biology and Biotechnology, Foundation for Research and Technology - Hellas, Heraklion, Crete, Greece

The manuscript aims to address the role of the proteasomal activity in aging and health span, using *Podospora anserina* as a model organism. The authors show that gene expression and protein abundance of proteasome subunits and proteasome assembly factor remain stable during aging and oxidative stress. The authors used strains overexpressing the catalytic proteasome subunits PaPRE2 and PaPRE3 to increase proteasomal activity, but found no difference in lifespan, suggesting that proteasome activity does not affect lifespan. The study would benefit by including proteasomal activity controls to confirm activity increase. This is particularly relevant since the positive effect of the proteasome in lifespan, senescence and healthspan has been convincingly demonstrated in other systems. The authors suggest that the reporter they use, GFP-Cl1, is processed by the autophagic machinery. It would be interesting to examine whether overexpression of this construct causes aggregation of GFP which in turn triggers autophagy that subsequently clears the aggregates which cannot be cleared by the proteasome. As the authors state in their discussion toxic aggregates could be formed and this possibility should be experimentally evaluated. For instance the authors should evaluate whether ERAD (endoplasmic reticulum associated protein degradation) takes place, since accumulation of protein aggregates could initiate ER stress and ERAD. It is known that under various stress conditions proteasomal activity is enhanced. Perhaps the authors should try other conditions in addition to oxidative stress (ER stress, heat stress etc.).

I have read this submission. I believe that I have an appropriate level of expertise to confirm that it is of an acceptable scientific standard.
